# Gingival cyst of the adult in a pediatric patient: Report of a case

**DOI:** 10.1002/ccr3.2646

**Published:** 2020-01-10

**Authors:** Julia Richman, Jeff Johnston

**Affiliations:** ^1^ Department of Pediatric Dentistry University of Washington Seattle Washington; ^2^ Department of Periodontology and Oral Medicine The University of Michigan School of Dentistry Ann Arbor Michigan

**Keywords:** child, cyst, gingival, Gingival Cyst of the Adult, lesion

## Abstract

Although the gingival cyst of the adult is considered rare in children, it can occur. The GCA can cause necrosis of the alveolar bone if untreated and should be considered in the differential diagnosis of raised gingival lesions.

## Graphical abstract

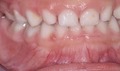

## 1 INTRODUCTION

Gingival cysts of adults (GCA) are thought to be a relatively uncommon developmental odontogenic cyst. However, they are not as rare as indicated by a review of the literature.^1^ The most common area of occurrence is the lower anterior region and the lower cuspid and first bicuspid.^2^ The GCA histologically is a true cyst presenting as 1‐3 layers of squamous to cuboidal cells with occasional glycogen‐filled clear cells. Most are unicystic and free of inflammation. These cysts are thought to be very rare in children. Buchner and Hansen reported a case in a 7 year old in 1979, and Park, Cheung, and Campbell published a case in a 4‐year‐old boy in 2017.^2^, ^3^ A review of 20 cases by Viveiros et al in 2019 agrees that although gingival cysts are rare, they should be considered in the differential diagnosis of any bluish asymptomatic nodule.^4^

Differential diagnosis includes fibroma, peripheral ossifying fibroma, giant cell granuloma, pyogenic granuloma, and periapical bone lesions.^5^ Excisional biopsy is the treatment of choice. Recurrence has been reported to be rare.

## 2 CASE REPORT

**Figure 1:**
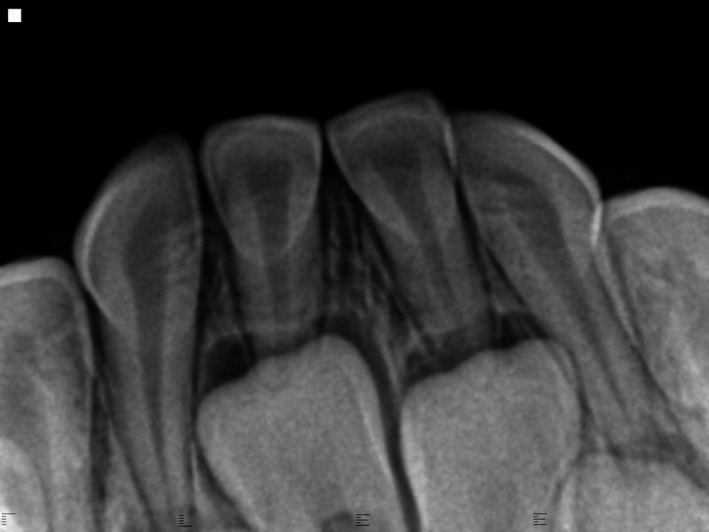
Preoperative radiograph of the region showing slight radiolucency mesial to tooth #R in the region of the gingival lesion

**Figure 2:**
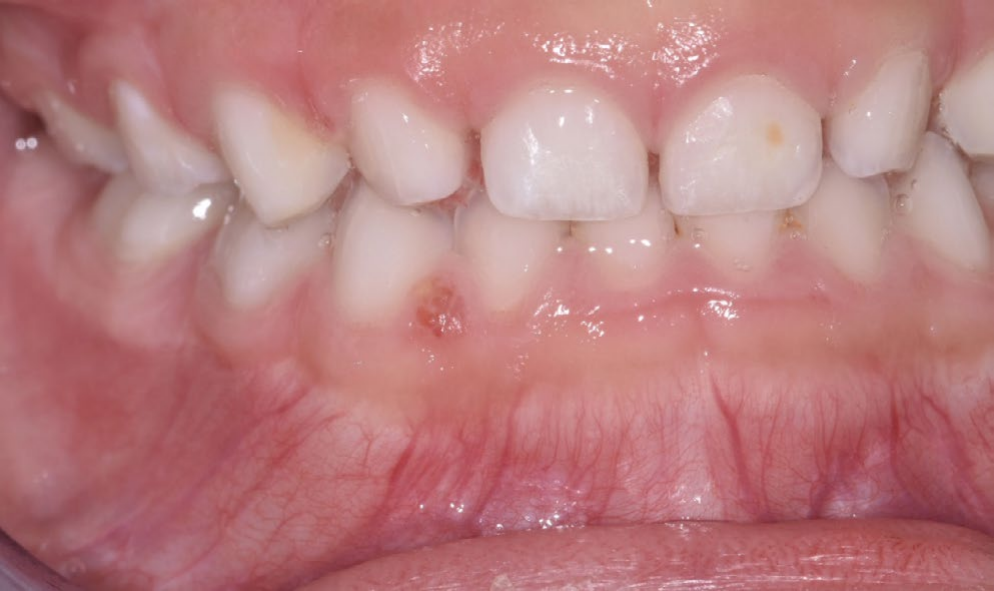
Preoperative photograph of gingival lesion between primary teeth #Q and #R

The patient, a healthy boy of Asian ethnicity, first presented to the dental clinic in August 2016 at the age of 3y10m. At his routine dental exam in August 2017, a “small inflamed area” was noted near tooth #Q (FDI #82). A periapical radiograph obtained that day showed a subtle radiolucency near the mesial aspect of the periodontal ligament space of tooth #Q (FDI #82) (Figure 1). The decision was made to monitor this area. It was noted again in March 2018 as possibly traumatic in origin. Later in March 2018, the patient was brought in for a limited examination for the “sore” near tooth #Q (FDI #82). Per the patient's father, the area had been very stable and he had noticed no change. It was noted that based on clinical appearance the lesion may have been a fibroma. At the next routine dental examination in September 2018, the lesion remained present and unchanged (Figure 2). At that point, a differential diagnosis of: gingival cyst, hemangioma, pyogenic granuloma, or peripheral giant cell granuloma was established. After discussion with the patient's father, the decision was made to take the child to the operating room for surgical excision and biopsy due to the need to reflect a mucoperiosteal flap to remove the lesion in its entirety, the child's young age, and the risk that cooperation would deteriorate during the procedure.

**Figure 3:**
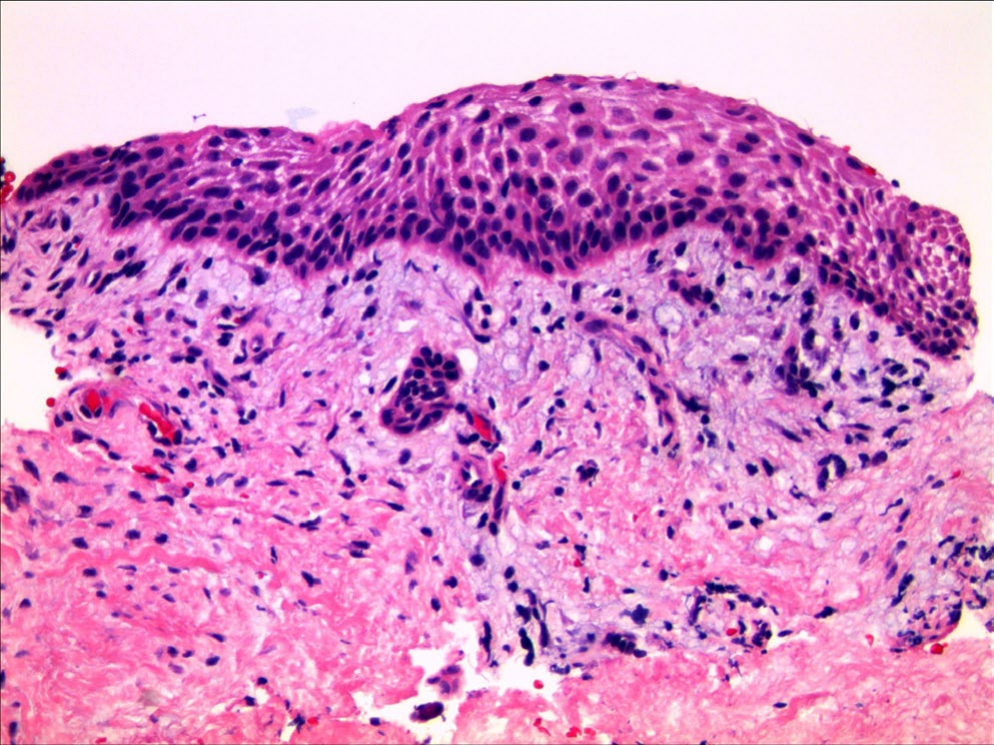
(20× H&E slide) The microscopic sections display a fibromyxoid connective tissue cyst wall lined by nonkeratinized squamous cystic epithelium. An odontogenic epithelial rest is present subjacent to the luminal surface. (Photograph courtesy Dr Mikelle Kernig.)

**Figure 4:**
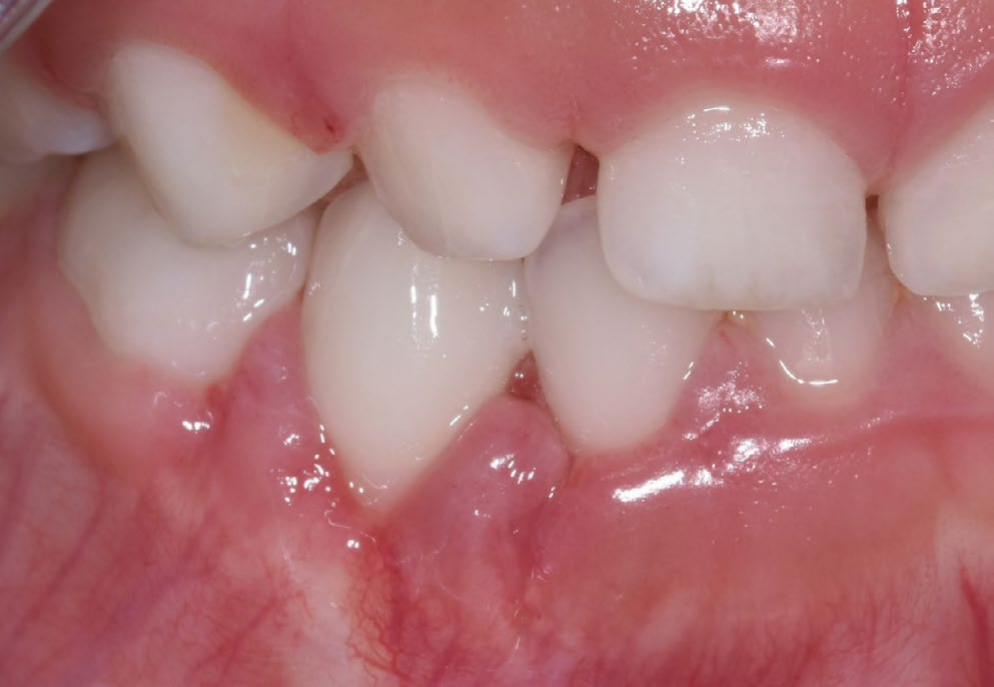
Postoperative photograph showing healing at 1 wk postsurgery

The patient was taken to the operating room in October 2018. General anesthesia was induced, a nasotracheal tube was placed and secured, and a throat pack was placed. About 1cc of 2% lidocaine with 1:100K epinephrine was infiltrated. A #15 scalpel was used to do a full‐thickness mucoperiosteal flap, which was elevated and reflected. The lesion was identified as a round well‐circumscribed (encapsulated) gray mass with a soft texture. The surface was smooth and extended into the cortical bone. The lesion was removed in two fragments and submitted for microscopic examination (Figure 3). The cortical bone was curetted. The surgical wound was closed with 1 horizontal mattress and 2 interrupted 3‐0 chromic gut sutures. The patient was extubated, monitored in PACU, and discharged uneventfully. He presented for postoperative evaluation one week later. Healing was uneventful and satisfactory (Figure 4).

The biopsy report noted:

Anterior right mandible, gingiva between deciduous teeth R and S, biopsy:—Consistent with developmental gingival cyst.

COMMENT: Although the patient is 5 years old, gingival cyst of the adult is considered in the differential diagnosis based on the given clinical presentation. The histologic features are nonspecific and could represent gingival cyst of the adult or an intraosseous developmental odontogenic cyst such as dentigerous cyst. Correlation with available imaging studies is encouraged. Evidence of a more aggressive odontogenic cyst, such as odontogenic keratocyst or cystic ameloblastoma, is not seen. Histologic sections demonstrate a small fragment of minimally inflamed fibrovascular connective tissue surfaced by nonkeratinizing cystic squamous epithelium. Immediately subjacent to the cystic epithelium, a single odontogenic epithelial rest is identified. In addition, a small fragment of dense fibrous connective tissue with associated gingival surface squamous epithelium is present.

The biopsy report made mention of teeth numbers R and S; however, this was likely a typographical error since the lesion was located between teeth numbers Q and R (FDI #82 and #83). A dentigerous cyst (the other lesion to be named on the differential diagnosis) is not very likely due to the unaggressive and stable nature of the lesion as demonstrated through chart notes.

The child continued to be a regular dental attender. The healing at approximately 10 months postsurgery was within normal limits. No recurrence was noted as of publication date.

## 3 DISCUSSION

Several etiologic possibilities of the GCA were discussed by Ritchey and Orban. These include heterotopic glandular elements or epithelial remnants or the enamel organ, periodontal ligament (membrane), or dental lamina. Epithelial remnants may also migrate from the surface epithelium as a degenerative epithelial peg or proliferation from trauma.^6^, ^7^ A distinction should be made between the gingival cyst and the lateral periodontal cyst on the basis of cell origin. Lateral periodontal cysts arise from the periodontal ligament.^8^, ^9^ However, the distinction has been contested by Wysocki and Brannon.^10^ Both cysts display glycogen‐rich clear‐cell rests of dental lamina, suggesting a histogenetic link between the GCA and the lateral periodontal cyst. There is also a coexistence of features such as enlargement and cystic degeneration with microcyst formation in the clear‐cell rests of dental lamina, focal thickenings (plaques) composed of clear cells in the epithelial lining, and a thin epithelial lining exhibiting varying numbers of clear cells. The WHO classifies these cysts as separate; however, both are considered developmental odontogenic cysts.^5^

Gingival cysts are generally <1 cm possibly due to the fact that they arise from postfunctional cells of the dental lamina. The prevalence of gingival cysts in adults has been reported as 0.3% of all odontogenic cysts.^11^ They are generally slow‐growing asymptomatic oval or round lesions in the attached gingiva. They may appear as radiolucent due to pressure atrophy of the subjacent alveolar bone. Less than 20% of cases described in the literature presented with a radiographic finding.^12^ Although these lesions are slow growing, excisional biopsy is the treatment of choice. If the lesion is not removed via excisional biopsy, it could potentially progress and cause pressure necrosis of the alveolar bone. Biopsy is also indicated to rule out other gingival lesions on a differential diagnosis. Since young children may not be able to tolerate an excisional biopsy in the dental setting, general anesthesia or sedation may be indicated.

This is the third reported case of a gingival cyst of the adult appearing in a pediatric patient. Although gingival cyst of the adult is thought to be rare in the pediatric population, it is prudent to consider gingival cyst in the differential diagnosis in a pediatric patient for gingival lesions that do not resolve spontaneously, especially those arising in the anterior mandible to first primary molar area.

## 4 SUMMARY

● Gingival cyst of the adult is rare in children but should be considered for raised gingival lesions in the anterior mandible.
● If untreated, a GCA may result in pressure necrosis of the alveolar bone.
● The GCA is best treated with excisional biopsy, and recurrence is rare.

## CONFLICT OF INTEREST

The views expressed in this article are those of the authors alone and do not represent the views of any academic institution or business. The authors do not have any conflicts of interest or financial disclosures or sources of funding to report.

## AUTHOR CONTRIBUTIONS

Dr Richman: provided case history, images, and surgical and clinical details. Dr Johnston: conducted a literature review and helped treatment plan the surgical approach. Both authors: contributed equally to the manuscript.
